# Hysterics and Neurasthenia Caused by Dental Irritation

**Published:** 1884-11

**Authors:** 

**Affiliations:** Physician in the Austrian Army


### Hysterics and Neurasthenia Caused by Dental Irritation.

BY DR. SICKINGER, PHYSICIAN IN THE AUSTRIAN ARMY.

Translated by Alma Fuellgraff, D.D.S.

In January of this year, a lady, Miss Ernestine F., came to my office for the purpose of having the upper central incisors filled. She began with the general complaint that she was very nervous whenever she needed the services of a dentist, and for this reason, Dr. Battlock, in Olmutz, had not been able to do anything to her teeth. Like all dentists, being used to such remarks, I did not attach much importance to them, but begged the lady to take a seat in the operating chair. Wishing to make a careful examination, I requested the lady to lean back in the chair, and then tried to divert her attention by talking on indifferent subjects. As soon, however, as the patient saw the half-hidden instrument, she became greatly agitated, although I assured her that she would experience little or no pain; at the same time I made some examination of the sound teeth, but the moment I tried to separate the teeth with the file she grew very nervous. I now thought it was time to begin the work, and commenced very cautiously to examine the cavity where I expected to find pulpitis. The very approach of the instrument apparently gave pain, consequently I endeavored to quiet the patient, but the agitation increased rapidly, and she trembled violently; then, as much as these symptoms diminished, the color of the face changed, she grew pale, and slight convulsions of the extremities occurred, followed by complete fainting. At the very beginning of the sitting I knew that I had to do with a neurasthetic patient, but I did not fully comprehend the extent of the difficulty. I was much interested in the case, and requested the lady to call again the next day. When she came, accompanied by her sister, I found that the patient, a robust lady of forty years of age, unmarried, had never had a severe illness, nor ever suffered from epilepsy. The nerve normal, the gums somewhat pale but not exactly anæmic. I was not able to ascertain any raising or diminishing of the sensibility of the skin, nor did I examine the electro-muscular contractability; patient claimed not to have had globus hystericus; she, as well as her sister, claimed to be perfectly well. However, when I inquired more closely I found that she also fainted in Dr. Battlock's office, in Olmutz, above referred to, without having her teeth treated at all. She related that she had fainted on one occasion from sticking herself with a needle, though the death and burial of her much-beloved mother had no such effect.

After much persuasion, I induced the patient to sit down on a chair, and the moment I touched the healthy teeth with a burnisher she declared that a fainting fit was coming on, which result, however, was prevented from the very fact that she was sitting in an ordinary chair, and nothing of importance could be done to her teeth.

The majority of patients go to the dentist in fear and trembling—and sometimes with reason. Fainting fits will now and then accompany difficult operations, yes, even slighter ones; however, such conditions are rare.

This case is remarkable from the fact that the patient made the greatest effort to overcome the symptoms, and is, according to my idea, a case of dental neurasthenia, or, if you like, dental hysterics. I think the latter is only an increased morbid process of the first. The absence of globus hystericus pleads, in this case, for neurasthenia; the slight convulsions, the acuteness and violence of the symptoms, for hysterics.

George M. Beard would call this neurasthenia. According to Hesse, Erb, and others, the globus hystericus and the frequent palpitations of the heart may be wanting in neurasthenia. The lack of these symptoms, however, gives considerable margin for differential diagnosis.

